# Bioethical Implications of Vulnerability and Politics for Healthcare in Ethiopia and The Ways Forward

**DOI:** 10.1007/s11673-022-10210-x

**Published:** 2022-09-22

**Authors:** Kirubel Manyazewal Mussie, Bernice Simone Elger, Mirgissa Kaba, Félix Pageau, Isabelle Wienand

**Affiliations:** 1 Institute for Biomedical Ethics, University of Basel, 4056 Basel, Switzerland; 2 Center for Legal Medicine, University of Geneva, 1205 Geneva, Switzerland; 3 School of Public Health, Addis Ababa University, 1230 Addis Ababa, Ethiopia; 4 Faculty of Medicine, Laval University, Quebec, G1V0A6 Canada

**Keywords:** Bioethics, Vulnerability, Gender, Health governance, Political instability, Ethiopia

## Abstract

Vulnerability and politics are among the relevant and key topics of discussion in the Ethiopian healthcare context. Attempts by the formal bioethics structure in Ethiopia to deliberate on ethical issues relating to vulnerability and politics in healthcare have been limited, even though the informal analysis of bioethical issues has been present in traditional Ethiopian communities. This is reflected in religion, social values, and local moral underpinnings. Thus, the aim of this paper is to discuss the bioethical implications of vulnerability and politics for healthcare in Ethiopia and to suggest possible ways forward. First, we will briefly introduce what has been done to develop bioethics as a field in Ethiopia and what gaps remain concerning its implementation in healthcare practice. This will give a context for our second and main task – analyzing the healthcare challenges in relation to vulnerability and politics and discussing their bioethical implications. In doing so, and since these two concepts are intrinsically broad, we demarcate their scope by focusing on specific issues such as poverty, gender, health governance, and armed conflicts. Lastly, we provide suggestions for the ways forward.

## Introduction

Vulnerability and politics are among the key topics of discussion in healthcare in general and in bioethics in particular. Ethical challenges that vulnerable population groups often experience in healthcare include, for example, health inequalities and discrimination (Boldt 2019). Factors determining patient vulnerability could be, among others, personal–such as age, gender identity, and socioeconomic conditions–and health system-related such as discriminatory procedures, fragmented care, and lack of resources (Ferreira et al. 2021). Similarly, the political situation of the context in which the healthcare system operates has important bioethical implications for the care patients receive. Common ethico-political challenges affecting healthcare delivery could include weak ethical health leadership, lack of governmental commitment to address health challenges specific to certain population groups, and armed conflicts (Pellegrino 2006; Hussein 2009). In the below sections, we operationalize these two concepts– as they are very broad– and, focusing on Ethiopia, discuss their bioethical implications for healthcare. To better contextualize their bioethical implications for healthcare practice in Ethiopia, we start by providing a summary of the development of bioethics in the country and its current state as a formal discipline.

### The Development of Bioethics in Ethiopia–A Synopsis

According to available work discussing the development of bioethics in Ethiopia (Addissie and Tesfaye 2014), the field of bioethics is new and one of the most understudied subjects in the country. The little knowledge that exists focuses on medical research and not on healthcare. However, a note of caution here is not to conclude that the existence of the notion of bioethics is as recent as the formal emergence and recognition of bioethics as a disciplinary field. Agreeing with Ten Have’s claim that informal bioethics was born together with the human species and has been functioning since then (Have 2013), we argue that the notion of bioethics was present in ancient communities in Ethiopia, reflected in religion, social values, and local moral underpinnings. In line with this, some writers argue that Indigenous moral principles such as, *inter alia*, “šine migibar” (ethical conduct) and living together with humankind and nature, are found in religious teachings and Indigenous political structures such as the Gadaa system of the Oromo (the largest ethnolinguistic group in Ethiopia) (Abera 2021; Milkias 1976; Kiros 1996). There are similar reports on the contribution of Indigenous moral principles to bioethics in other African countries such as Tanzania where bioethics as a field is believed to be young (Chuwa 2014).

Even though the notion of bioethics was apparent in traditional and Indigenous moral values, the formal recognition and analysis of bioethical issues are believed to have started together with the introduction of Western biomedicine when Emperor Menelik II (1889-1913) and Emperor Haile Sellassie I (1930-74) involved foreigners to advance health services in Ethiopia in the early twentieth century (Addissie and Tesfaye 2014; Kloos 1998b). Since then, regardless of the ongoing tensions in harmonizing Western with Indigenous moral values (Milkias 2008; Merawi and Kenaw 2019), different stakeholders such as academic institutions and the Ministry of Health have been facilitating the development of bioethics as a field of study. However, “there is a long way to go before ethics education, professional development and ethical support systems (such as clinical ethics committees) are in place” (Miljeteig et al. 2017a, b, 6).

Formal knowledge of bioethics is generally deemed inadequate in Ethiopia. In 2017 medical ethics was part of the curriculum at all twenty-eight universities that offer medical training in Ethiopia, however students were receiving very little or no ethics education and there was a shortage of medical ethics teachers (Miljeteig et al. 2017a, b). Tiruneh and Ayele elaborate how the lack of deeper understanding of medical ethics among medical doctors in Ethiopia contributes to poor practice of code of ethics:

Even though, 459 (91.8%) of medical doctors took medical ethics course during medical education, the time (two credit hours) was not enough to deliver the course and integrate with the medical practice. Moreover, the course did not include the country’s Health Professionals’ Code of Ethics. This finding indicates that medical doctors lacked theoretical and practical aspect of code of ethics in medical practice. This might have contributed for the witnessed poor practice of code of ethics among medical doctors. (Tiruneh and Ayele 2018, 14)

Moreover, there is limited availability of graduating programs and scholars in bioethics (Addissie and Tesfaye 2014, 1129; Miljeteig et al. 2017a, b). This lack of medical ethics knowledge is reported to have serious consequences. There have been complaints from patients regarding medical treatments from physicians resulting from, among other things, a lack of adequate medical ethics knowledge (Wamisho et al. 2015; Abeje et al. 2019).

In the same vein, there have also been efforts from the government and professional associations to develop ethical guidelines to guide the ethical conduct of health practitioners (see Table 1).

**Table 1:** Available Ethical Guidelines for Health Practitioners in Ethiopia.

**Guidelines**	**Developer**	**Year**
Professional Code of Ethics and Conduct for Midwives	Ethiopian Midwives Association	2011
Health management, ethics, and research; blended learning module for the health extension program, both professional ethics for health extension workers and guidance for research in primary healthcare	Federal Ministry of Health	2011
Medical Ethics for Physicians in Ethiopia	Ethiopian Medical Association	2010
Code of Ethics For Medical Laboratory Technologists Practicing In Ethiopia	Ethiopian Medical Laboratory Association	2008
Introduction to Professional Nursing and Ethics: Lecture Notes for Professional Nursing Students	Ethiopian Public Health Training Initiative	2005
Health Ethics and Health Laws: Lecture Notes For Health Extension Trainees in Ethiopia	Ethiopian Public Health Training Initiative	2004

Source: Addissie, A., & Tesfaye, M. (2014). Ethiopia. In H. A. M. J. ten Have & B. Gordijn (Eds.), *Handbook of Global Bioethics* (pp. 1121-1139).

Such ethical instruments are crucial in guiding the medical practice of health professionals who face ethical dilemmas while, for instance, handling complicated health cases (Miljeteig et al. 2017a, b; Johansson 2008). There are more recent developments to improve the bioethics infrastructure. For example, since the establishment of the first ethics centre in Ethiopia in 2017–the Addis Centre for Ethics and Priority Setting–concerns for the integration of bioethics in health policies, programs and research have increased (Miljeteig et al. 2017a, b). However, more efforts to advance the field remain crucial. Providing additional comprehensive analysis of the challenges in advancing bioethics in Ethiopia and suggesting improvements in the overall trajectory of the field are broad and ambitious tasks that are beyond the scope of this paper. However, providing a summary of the development of bioethics in Ethiopia was determined necessary to give a context for the two concepts–vulnerability and politics–which are operationalized and discussed below.

## Bioethical Implications of Vulnerability and Politics for Healthcare

The aim of this paper is to discuss the bioethical implications of vulnerability and politics for healthcare in Ethiopia and to suggest possible ways forward. There are three points to consider in relation to this objective. First, the two concepts this paper aims to address are broad and we only discuss some ethical aspects most relevant for healthcare in Ethiopia. Second, the choice of these topics depended on the following reasons: (a) to be specific, (b) to integrate the in-depth experiences of two of the authors (KMM, MK), and (c) because these topics, driven by the ongoing pressures from poverty, gender disparities and armed conflicts, are one of the most pressing issues currently influencing healthcare in Ethiopia (Kaba et al. 2020; Borde et al. 2022; Tesema and Kinfu 2021). The third point is that the focus of this paper is ethical issues relating to healthcare provision and not to medical research. This is because healthcare ethics is relatively less investigated whereas, though still limited, there have been efforts to advance knowledge on research ethics (Addissie and Tesfaye 2014; Feleke et al. 2015; Miljeteig et al. 2019). According to Addissie and Tesfaye’s report on the development of bioethics in Ethiopia, major and current bioethical issues in healthcare warranting further analysis include, *inter alia*, end of life, autonomy and disclosure, healthcare system and access to healthcare, reproductive health, traditional medicine, medical malpractice, ethics of public health and medical emergencies, and abortion (Addissie and Tesfaye 2014). These ethical issues are also echoed in empirical studies carried out in Ethiopia (Abadiga et al. 2019; Addissie and Tesfaye 2014; Alem 2002; Defaye et al. 2019; Beyene 1992; Miljeteig et al. 2017a, b). Thus, in what follows, we will examine two challenges–vulnerability and political instability–and their bioethical implications for healthcare practice in Ethiopia. Our description is by no means comprehensive but, as we come from different backgrounds such as bioethics, medicine, public health, social sciences, and philosophy, it mirrors our multi-disciplinary perspectives on bioethical issues. Literature suggests that analysis of social, institutional, and economic factors in the development of bioethics is worth discussing in the context of other countries such as, for example, the United Kingdom and the United States (Chadwick and Wilson 2018; Scher and Kozlowska 2018). Thus, we believe that doing the same would benefit current discussions about healthcare ethics in Ethiopia.

### Vulnerability

Vulnerability is a crucial and complex concept in bioethics, regardless of whether it is used in the context of research or healthcare (Boldt 2019). Attempting to thoroughly discuss this complex concept falls outside the scope of this paper. However, Boldt’s definition of vulnerability–“a state of physical, emotional, and cognitive stability that is in danger of being disturbed or destroyed due to being susceptible to destabilizing influences” (Boldt 2019, 2)–is suitable for the discussion that follows since we will highlight in it two factors (poverty and gender dynamics) that can compromise peoples’ wellbeing. Vulnerability is a bioethical issue because vulnerable people are subject to harm and/or exploitation (Macklin 2003). The application of vulnerability is mostly confined to clinical research and there is a wide gap in understanding its relevance for healthcare (Boldt 2019). Thus, in what follows, we will focus on vulnerability in the context of healthcare provision and utilization. Several studies indicate that human health vulnerability is a serious challenge in sub-Saharan Africa where wider socioeconomic and political factors put many lives at risk (Morudu and Kollamparambil 2020; Atake 2018; Wamoyi et al. 2014), and this applies to Ethiopia as well.

### Poverty as Determinant of Vulnerability

There are different determinants of vulnerability in the Ethiopian context and one is poverty. Poverty, especially when coupled with diseases, is a dehumanizing factor: “the vicious circle of poverty and disease, which dehumanize the poor, is doubly unethical” (Chuwa 2014, 7). There exists a strong bond between vulnerability and poverty (Gallardo 2018) and discussing this link in Ethiopia–a resource-scarce country–appears both relevant and important. A dominant topic in empirical studies that are identified as typical for the Ethiopian bioethical context is resource scarcity (Ali 2014; Gebremariam et al. 2018; Maes 2012; Sagbakken et al. 2013).

Poverty poses an ethical concern for both patients and healthcare providers. For patients, it increases vulnerability and weakens their power to enjoy equal health rights and exercise autonomy (Newdick 2017; Ribeiro and Zoboli 2007). The main ethical question in this context is how healthcare systems should distribute healthcare resources fairly in a way that empowers poor patients and promotes patient autonomy. Shrime and colleagues underline that “distribution of benefits is most equitable, however, when non-medical costs of care, such as transportation, food, and lodging, are no longer shouldered by patients” (Shrime et al. 2016, 9). Also, for healthcare providers, scarcity of resources results in ethical dilemmas. Miljeteig and colleagues state that in resource-limited settings, the most frequently encountered ethical dilemmas relate to the allocation of resources whereas biomedical ethics in high-income countries aches from “futility discussions and conflicts about autonomy” (Miljeteig et al. 2019, 1). Similarly, other studies also show that Ethiopian healthcare providers frequently encounter scarcity-related dilemmas, and this caused them regret about choosing their profession (Defaye et al. 2015, 2019; Miljeteig et al. 2017a, b). This is exacerbated by the absence of clinical ethics committees in most hospitals to support health professionals dealing with such dilemmas (Miljeteig et al. 2017a, b).

Deterioration of health and wellbeing among older adults in Ethiopia is a relevant example to demonstrate the role of poverty in increasing vulnerability among specific population groups. The growth of the aging population in Ethiopia, combined with the country’s rapid change in terms of culture and economy, gives rise to ethical concerns not only in the ways healthcare providers give services but also in how administrations and policies address aged care. Although traditional family and community support are believed to be the main sources of care for older people, these sources are weakening mainly due to the growth of industrialization and urbanization (Lemma 2014; Teka and Adamek 2014). As a result, a significant number of older adults “end up begging in the streets or living in destitute condition around places of worship” in the main urban cities (HelpAge International 2013, xiii). Alambo and Yimam studied the social support system in the Gedeo zone of southern Ethiopia–a rural setting–and described the situation as follows:

The long-standing social values, norms, and belief systems of the ethnic group have always prescribed families, extended families, and neighbors to respect and take care of their older members. Nonetheless, poverty in rural households, the diffusion of urban values, and the incipient erosion of longstanding rural values, as well as the tendency of rural youth to abandon agriculture are challenging the capacity of community members to ensure sustained availability of adequate informal care for older adults. (Alambo and Yimam 2019, 15)

Infectious diseases linked to poverty are also examples of how poverty increases vulnerability among groups, creating particular ethical challenges in relation to autonomy and justice. In this regard, for example, tuberculosis (TB)–an airborne disease that evidence refers to as a disease of poverty (The Lancet 2005; Mussie et al. 2021, 2019)–further illuminates the link between poverty and bioethics. TB is intertwined with larger socioeconomic and political factors that complicate its management and control (Moonan et al. 2011; Mussie et al. 2020; Manyazewal et al. 2020a, b) and thus, is among the poverty-driven infectious diseases that are topics of biomedical ethics and human rights. This is especially the case when it comes to TB treatment: particularly the internationally recommended treatment strategy called DOTS (directly observed treatment, short-course), which involves treatment of TB patients with first-line TB drugs for 6-9 months. One component of this strategy is DOT (directly observed treatment), which aims to ensure that TB patients visit TB clinics daily and take TB drugs in the needed dosage and time, with the presence and supervision of a health professional.

The DOT strategy is strongly criticized for interfering with principles of bioethics. Due to its paternalistic approach, DOT can be seen as “an intrusion upon autonomy” (Bojorquez et al. 2018, 805). In Ethiopia, several studies (Mussie et al. 2020; Sagbakken et al. 2008, 2013) further illuminate this discourse about DOT and patient autonomy and suggest that this TB treatment strategy compromises patient independence and autonomy as it comes with stressful obligations such as visiting TB clinics daily and swallow drugs while being observed by a health professional. In addition to autonomy, DOT is also reported to conflict with the principles of beneficence and non-maleficence. The following quote from a comparative TB study conducted in Ethiopia and Norway summarises the ethical concerns as follows:

It is difficult to see that DOT is effective or doing more good than harm when patients who interrupt treatment risk being excluded from further treatment and follow-up because they cannot adjust to the rules of the system. We also question whether the potential benefits of DOT outweigh negative features if patients are denied the opportunity to optimize their health while receiving treatment. Similarly, we ask whether a measure such as DOT is doing more harm than good in the long run, if patients lose their jobs or social positions. (Sagbakken et al. 2013, 6)

In the above qualitative study of DOT practices in Ethiopia and Norway, Sagbakken and colleagues indicate that DOT itself influences healthcare providers to thoughtlessly and carelessly treat patients and inflict harm upon them (Sagbakken et al. 2013). As the purpose of DOT is to ensure that patients take drugs, it is easy to overlook the living conditions and perspectives of the patient unless the healthcare provider chooses to engage more and empathize with the patient. The insensitive aspects of TB treatment procedures and, more broadly, the inadequate global health efforts to advance TB treatment, raise ethical and human rights concerns in Ethiopia and beyond on which policymakers need to act (World Health 2017, 48; Chuwa 2014).

### Gender Dynamics as Determinant of Vulnerability

In addition to poverty, gender is another determinant of vulnerability we discuss in this paper. However, it is important to note that gender per se cannot and should not be considered as a direct factor for vulnerability and that this is the premise upon which our discussion is based. Nevertheless, gender can play an indirect role in facilitating vulnerability when, for example, society uses it as the basis of prejudice and discrimination. In line with this, empirical studies in Ethiopia show that women in some contexts would experience health vulnerabilities. Elaborating on this challenge, one study that reported on gender, health, and women empowerment in Ethiopia states that “the low status of women prevents them from recognizing and voicing their concerns about health needs” and that “the low status of women and their disempowerment is highly associated with poor health outcomes” (Lailulo et al. 2015, 1). As compared with urban cities, gender inequality is a more serious phenomenon in the rural parts of Ethiopia where nearly eighty per cent of the total population resides (OECD and Policy Studies Institute 2020). This difference between the two parts of the country can be attributed to differences in terms of women empowerment and gender awareness, as well as the differences in health education and health service provision. For example, women’s autonomy over their health is reported to be much higher in urban areas as compared with rural (Tarekegn et al. 2014; Tiruneh et al. 2017), and “women in urban areas were more likely than rural women to receive reproductive health care” (Wado 2018, 741).

Maternal healthcare is one area that can demonstrate the gender gap in healthcare and its impacts on female patients in Ethiopia. Even though Ethiopia in general has low national health service coverage (34.3%) and utilization (Eregata et al. 2019), evidence shows that women can be affected more by this challenge. Several studies report that both provision and use of maternal health services in Ethiopia are at a low level (Tiruneh et al. 2017; Tarekegn et al. 2014; Kiross et al. 2021). Giving a more ethical perspective to this discussion, several studies (Tarekegn et al. 2014; Tiruneh et al. 2017, 2021; Alemayehu and Meskele 2017; Tadele et al. 2019; Tesema et al. 2021) elaborate on the limits of women’s autonomy in Ethiopia and how that interferes with their health and wellbeing. For example, one study–which concludes that “improving women’s autonomy will help to attain both gender equality and improved use of health services” (Wado 2018, 729)–emphasizes that women’s inability to make decisions about their health is linked with poor health among themselves and their children. This confirms that the negative consequences of low female autonomy also affect child health. Evidence shows that illness and mortality among children below five years of age are strongly linked with compromised maternal health (Zewudie et al. 2020) which, as shown above, is partly influenced by the lower socioeconomic status and autonomy of women. An ethnographic study further adds that limited decision-making power among women has implications for their health and suggests that “women’s lack of autonomy in making key decisions to live the life they wish to live implies that they have not achieved empowerment” (Mengistie 2022, 12). However, in contrast, a cross-sectional study conducted in southern Ethiopia concludes that women who are younger, have a higher level of formal education, and have better occupational status have a high decision-making power as compared to those women without these characteristics (Belay et al. 2016). Without disregarding the different perspectives these studies give, an important implication shared in existing evidence is that gender inequalities affecting women could be seen as social in nature. The major cause for lower female autonomy in Ethiopia might be more cultural than economic; “increasing the access of economic resources alone is insufficient but requires increased women’s decision-making and control of household resources” (Tiruneh et al. 2021, 2). One study summarizes the situation of women as follows:

Ethiopian societies are socialized in a way whereby women have a lower social status compared to men. From a young age, autonomy is promoted among boys, while girls are not given any autonomy, but they are trained to be more obedient and dependent, and to household chores such as cooking, in preparation for marriage and caring for their households in later years. (Lailulo et al. 2015, 2)

The gender-related accounts given above imply the presence of ethical issues relating to healthcare, such as limited access to healthcare services, health inequality, and lack of health decision-making autonomy. Bioethics deals with gender-related issues, and, in support of this claim, literature demonstrates a strong link between bioethics and gender (Glover 1992; Crosthwaite 2009). To elaborate on this, the following passage from Crosthwaite’s work is worth quoting at length:

[…] women’s most frequent complaints concern failures of respect: not being taken seriously as authorities on their own experience and preferences; not being properly informed about their condition and treatment options; and generally not being accorded the rights of competent adults to decide about their own health care. Gender stereotypes and behavior patterns foster these failures and reinforce the inequalities inherent in physician–patient encounters. Yet while bioethics has been centrally concerned with issues of patient autonomy and power imbalances between health-care professionals and their clients, it has had relatively little to say about the impact of gender on these issues. (Crosthwaite 2009, 38)

#### Fragile Political Situation

Ethiopia often experiences political instabilities that have ethical implications for healthcare provision in the country. The consequences of a weak political situation have affected not only major sectors such as education and the economy, but have extended to the health system. However, this is not to disregard success stories in health that were the results of a conducive political environment. For example, “Ethiopia’s expansion of primary healthcare over the past 15 years [2005 - 2020] has been hailed as a model in sub-Saharan Africa”, which is “attributed to strong leadership and ‘political will’” (Croke 2020, 1). In addition, there have been positive changes through political will from high-level government authorities to employ a bottom-up approach by involving community-level stakeholders (Admasu 2016). Despite these, however, the country’s health system has not benefited much from any of the major governmental systems since the early twentieth century. For instance, primary healthcare has been inadequately available to Ethiopians throughout the last three political systems–monarchy, socialism, and democracy–as a result of, *inter alia*, weak political structures (Kloos 1998a). There is a connection between politics and bioethics (Pellegrino 2006) and below, we further illustrate this link in Ethiopia by referring to ethical challenges with healthcare leadership and armed conflicts.

### Challenges with Healthcare Leadership

The concept of ethical health leadership has transformed from a traditional and narrow focus on the behaviors of the health practitioner in patient-provider relationships to a contemporary and broader focus on behaviors and practices in the organization of a health system. Thus, in this section, we adopt Ho and Pinney’s conception of ethical health leadership: “as care becomes increasingly integrated and population-based, a broader systematic approach to ethical healthcare leadership is required” and thus, in today’s complex and predominantly integrated health provision, “ethical healthcare delivery requires managers, executives, and even trustees and policy-makers to be held accountable not only for how individual care encounters are conducted but also for how the system is organized to ensure quality care within and across defined populations” (Ho and Pinney 2016, 39).

The World Health Organization (WHO) asserts that leadership and governance is one of the building blocks of a strong health system that is responsive to public health needs (WHO 2007). Similarly, the Federal Ministry of Health (2015) of Ethiopia underscores political leadership and governance as one of the important pillars of an effective health system. Lack of good governance as manifested through, for example, corruption and mismanagement of health resources, is most responsible for public health challenges such as health inequalities in low and middle-income countries (García 2019; Munezhi and Hammad 2021). Ethiopia is not an exception.

There are some gaps in healthcare governance that interfere with the fair and proper delivery of healthcare services in Ethiopia. For example, there are records of efforts to shift health resource allocation from urban to rural areas because urban ideologies challenged the ruling party’s political ideologies (Croke 2020). Moreover, as one study reports, lack of good governance as manifested through corruption and poor quality of healthcare regulatory bodies is one of the major factors associated with child undernutrition (Biadgilign et al. 2019). Such challenges in healthcare governance are also reported as one of the factors for another ethical issue in healthcare: medication error. A recent systematic review of medication error reports that at least one out of two medications are incorrectly prescribed, making medication errors very common in Ethiopian hospitals (Endalamaw et al. 2020). Further elaborating on the prevalence of medical errors and highlighting the gravity of the problem, the review reports the following:

The overall prevalence of medication error in Ethiopia was 57.6%. The pooled burden of medication administration and prescription error was 58.4% and 55.8%, respectively. Omission error (38%), wrong dose (38.5%), and the wrong combination of drugs (28.7%) were highly reported types of prescription errors, whereas missed doses (57.0%), technical errors (47.0%), wrong time (35.0%), and wrong dose (30.0%) were frequently observed medication administration errors. (Endalamaw et al. 2020, 3)

In Ethiopia, medical error affects many lives and different vulnerable population groups including older adults (Abegaz et al. 2018; Getachew et al. 2016) and children (Dedefo et al. 2016). Empirical evidence from Ethiopia states that there are several health governance-related factors contributing to medication errors, which include longer hospital stays (Abegaz et al. 2018), lack of medication preparation guidance (Baraki et al. 2018), and inadequate staffing and documentation systems (Alemu et al. 2017).

An additional challenge resulting from such gaps in healthcare leadership is the difficulty to ensure effective healthcare workforce management. More specifically, the underfunding and low recognition of professional and nonprofessional healthcare providers pose challenges to the effective delivery of quality healthcare. Several empirical studies report on economic challenges among healthcare providers in Ethiopia and how that affects the care they provide. For example, a recent qualitative study reports that low compensation among the participating health professionals had implications on the quality of healthcare they provided for tuberculosis patients (Mussie et al. 2021). Related challenges were also recorded in ethnographic studies that captured the experiences of nonprofessional, voluntary healthcare providers working in complex political-economic contexts in Ethiopia. For example, one ethnographic work investigating challenges in healthcare voluntarism in Addis Ababa, Ethiopia’s capital, reports that economic insecurity among voluntary community health workers affected their motivation to care for HIV/AIDS patients (Maes 2012). Similarly, Jackson et al. (2019) give a gendered perspective on this challenge and, in line with a systematic review reporting on articles evaluating the implementation of the health extension program (Assefa et al. 2019), summarize the experiences of women health extension workers (HEWs) as follows:

HEWs did not always have days off like civil servants such as Agricultural Extension Workers. They were often disturbed at night, on weekends and holidays, when women were giving birth, wanting access to family planning and for other emergencies. […] Many HEWs were unhappy with the lack of career path or opportunities to move into different or higher positions with better working conditions. They all wanted more training, both to upgrade their knowledge and improve their performance, and to progress into positions of higher status and responsibility which attract better salaries and conditions. (p. 311)

Thus, as the above empirical examples commonly emphasize, economic challenges among both care providers and receivers could imply a lack of access to quality care among the care receivers, which is an ethical concern in healthcare (Galarneau 1998; Cribb et al. 2020).

The strength of ethical health leadership determines the quality of healthcare delivery. The following quote from a review on the effect of weak governance on healthcare delivery in Asia further illuminates how gaps in ethical health leadership in low and middle-income countries affect healthcare provision and necessitate stronger attention from responsible bodies.

Common types of corruption like informal payments, bribery, and absenteeism identified in the review have largely financial factors as the underlying cause. Poor salary and benefits, poor incentives and motivation, and poor governance have a damaging impact on health outcomes and the quality of health care services. These result in high out-of-pocket expenditure, erosion of trust in the system, and reduced service utilization. Implementing regulations remain constrained not only due to lack of institutional capacity but also political commitment. Lack of good governance encourages frontline health care providers to bend the rules of law and make centrally designed anti-corruption measures largely in-effective. (Naher et al. 2020, 1)

Similarly, such gaps in health leadership in Ethiopia contribute to limiting the advancement of quality healthcare. One study reporting on the relationship between health governance and child health in Ethiopia highlights the gaps in good health governance and suggests that improving good governance by reducing corruption and maladministration can improve the efficient use of resources in the health sector (Biadgilign et al. 2019).

### Wars and Armed Conflicts

Wars and armed conflicts involve, among other things, the deliberate injuring or killing of people. The two world wars are good examples of the link between ethics and wars (Michael 2004). In addition to the ethical issues associated with warfare, there are other dilemmas such as the delivery of health services to wounded adversaries (Rosner 2010). As a country that often experiences civil wars, Ethiopia faces ethical challenges related to armed conflict (Admasu 2016, 183). Studies have recordd traditional forms of mediation and conflict resolution based on local moral values. For example, one ethnographic study investigating the tradition of conflict resolution in the Afar region of Ethiopia reports that “there are assemblies run by council of elders representing different clans in north Afar selected on the basis of age, wisdom, honesty and proper knowledge of local conditions” and that “inter-ethnic conflicts were better addressed by the indigenous institutions because of their participatory, transparent and flexible nature” while government legal institutions such as courts play a facilitating role to complement these traditional structures (Tafere 2013, 57). Similarly, other qualitative studies (Tenaw 2016; Mengstie 2022; Genet 2021) conducted in other regions of the country report the benefits of traditional structures in preventing and resolving intra-ethnic conflicts and suggest that government institutions collaborate with and strengthen such traditional systems. A full discussion of Indigenous conflict resolution mechanisms lies beyond the scope of this paper, however this brief summary of the roles of traditional structures provide an indication of the relevance of local moral underpinnings to address armed conflicts and their consequences on healthcare in Ethiopia.

There is limited evidence to show whether there exist formal bioethics structures in Ethiopia deliberating on bioethical issues related to armed conflicts and their consequences on healthcare. In Ethiopia, a significant public health challenge caused by armed conflicts and warranting ethical consideration is the weakening of the health system, which further leads to health inequalities. A review reporting on political leadership and health system in Ethiopia summarizes this as follows:

Ethiopia’s tumultuous history of conflict, war, and famine over the past half century has, among other things, undermined many of the attempts of past governments to improve the health of the Ethiopian population. There has been a struggle to achieve equity in the health system. (Admasu 2016, 183)

The recent conflict in northern Ethiopia, which started in November 2020, is also one relevant example for this discussion. The war caused massive internal displacements and the emigration of more than 70,000 civilians to neighbouring countries (Tesema and Kinfu 2021). From an ethical point of view, this is highly problematic as “forced migration creates further physical and mental health problems during transit, in enforced encampment, and because of restricted entitlement to health care in countries hosting refugees” (Razum et al. 2019, 1613). It is also reported that this war caused nearly ninety per cent of the region’s population to need immediate humanitarian assistance (World Food Programme 2021). There was also evidence of rape and sexual violence of women and girls during the northern Ethiopian war (United Nations 2021), requiring access to physical and mental healthcare and social supports. The bombing of health facilities has left many without hope of getting timely medical attention. The devastating effects of the Ethiopian war economy are one of the major impediments to improving the country’s health system (Kloos 1998a). In addition to the bioethical implications of the destruction of health systems are the ethical repercussions of the process of restoring and rebuilding health systems in the aftermath of wars. The process of restoring a war-stricken health system is a lengthy process, which leaves the health of many people in danger long after the armed conflict may have ended. There is also evidence of women and girls being subjected to rape and sexual violence by fighting forces during the northern Ethiopian war (United Nations 2021). Survivors’ access to necessary medical care has been hindered in part by the destruction of healthcare facilities and the limitations of humanitarian aid (Amnesty International 2021). Armed conflicts have intergenerational effects such as mental illness, infection, and malnutrition (Devakumar et al. 2014), which can characterise the health status and needs of people in the region.

## Conclusions and Looking Ahead

In this article, we discussed the challenges that vulnerability and unfavorable political conditions pose to healthcare provision and their bioethical implications. Vulnerability is a widely discussed topic in the literature and it illuminates major ethical concerns in healthcare including fairness, justice, and patient autonomy. Exacerbated by poverty and gender dynamics, vulnerability points toward critical ethical challenges in healthcare provision as well as health budgeting. In addition, the fragile political situation in Ethiopia also contributes to ethically problematic situations at both micro and macro levels. Ranging from gaps in health leadership that result in mistreatment of patients and medication errors, to wars and conflicts the results of which include stringent health budget allocation strategies and prioritization procedures of population groups, the different forms of the political situation in Ethiopia warrant serious attention from bioethicists and health policymakers.

In the foreseeable future, the healthcare system in Ethiopia could face further socioeconomic and political challenges which will require better engagement from different stakeholders. There are opportunities for moving forward. Abolishing larger structural factors such as poverty and political instability may not be easily achievable in the near future. While it is beyond the scope of this paper to present a detailed strategy to address these challenges, we propose a few steps based on our analysis. The first is to further the analysis of ethical challenges relating to vulnerability and political barriers in healthcare. Our discussion has touched on just a few aspects of these broad concepts and expanding these discussions is imperative among the scientific and research community. Furthermore, bringing these discussions to the community using community forums and the media could have more benefits. Efforts could focus on facilitating opportunities where experts and the community at large could exchange ideas and reflect on social values relevant to bioethics.

Secondly, and in addition to increasing bioethical knowledge on vulnerability and politics, more efforts could focus on practical steps to address the healthcare challenges relating to the two issues. For example, the Ethiopian government could improve the health workforce funding, particularly by focusing on health extension and community health programs. While more attention from conventional governmental institutions on conflict and vulnerability is important, there should also be efforts to engage traditional structures as part of the solution for these challenges. For example, the inclusion of traditional authority, elders, and women's associations could be used to expand the knowledge about treatment and ethical considerations locally. In addition, traditional forms of mediation and inquiry could be utilized in areas of conflict and those prone to vulnerability. The task of addressing the political challenges for healthcare, however, needs to be carried out with great caution. It is essential that any efforts to strengthen the congruence between politics and bioethics in Ethiopia do not appear a great threat to political leaders. In his work based on experiences from a neighbouring country, Sudan, Hussein (2009, 3) suggests: “bioethics is better started in the levels least-annoying to politicians, namely the doctor-patient, and the researcher-participant relationships” (2009, 3). We also suggest that bioethics might need to face and work with the challenging political situation in Ethiopia without clashing with politicians.

Our final set of suggestions is broader in that it is aimed at mainstreaming bioethics in both academia and policy. As established earlier, there is limited evidence of formal engagement with bioethical issues relating to conflict and vulnerability in Ethiopia. Thus, in the line of academia, it will be helpful to develop an awareness of bioethical issues through embedded ethics teaching. Recent developments to establish graduate programs in bioethics-related fields should advance. Increasing and strengthening bioethics graduate programs will, among other things: (1) address the shortage of bioethics scholars in the country, (2) further facilitate integration and analysis of bioethical inquiries in other fields in medicine and life sciences, and (3) facilitate the provision of more medical ethics training for healthcare providers. As a relatively new discipline that is as yet far from being broadly accepted in critical areas such as academia and (public health) policy, bioethics in Ethiopia needs to further develop its theoretical approach as well as implement concrete professional guidance for healthcare workers and policymakers. The Ministry of Health, in collaboration with other authorities in education and research, could improve both the quantity and comprehensiveness of ethical guidelines, which will further assist health professionals who deal with ethical dilemmas resulting from, for example, poverty and challenging health leadership. Healthcare authorities and ethicists could develop practical guidelines on how healthcare personnel and policymakers should react to these and other related present challenges.
